# Focused cardiac ultrasound conducted by neurologists in patients with stroke: A validation study

**DOI:** 10.1093/esj/23969873251345374

**Published:** 2026-01-01

**Authors:** Jorge Pagola, Piergiorgio Lochner, Radim Licenik, Giulio Maria Fiore, Felipe A Montellano, Victor Gonzalez, Valérie Pavlicek, Juan Alvarez-Cienfuegos, Sergio Moral, Roberto Muñoz Arrondo, Alberto Vera, Angel Ruiz, Jesús González Mirelis, Jorge Rodríguez-Pardo, Esther Pérez-David, Juan Manuel García-Sánchez, Lara Ruiz Gómez, Laura Amaya Pascasio, Elvira Carrión Ríos, Tania Rodriguez-Ares, Charigan Abou, María Payá, Laura Guerra, Ana de Arce, Ainhoa Benegas Arostegui, Muhammad Khaled Hasan, Vlatka Reskovic

**Affiliations:** Vall d’Hebron University Hospital, Stroke Unit, Neurology Department, Barcelona, Spain; Faculty of Medicine, Saarland University Hospital and Saarland University, Homburg, Saarland, Germany; North West Anglia NHS Foundation Trust, Neurology and Cardiology Department, Edith Cavell Campus, Peterborough, Cambridgeshire, UK; Vall d’Hebron University Hospital, Stroke Unit, Neurology Department, Barcelona, Spain; Department of Neurosciences, Psychology, Drug Research and Child Health (NEUROFARBA), University of Florence, Florence, Italy; Stroke Unit, University Hospital Careggi, Florence, Tuscany, Italy; Institute of Clinical Epidemiology and Biometry, Julius-Maximilians-Universität Würzburg, Würzburg, Germany; Department of Neurology, University Hospital Würzburg, Würzburg, Germany; Cardiology Department, Vall d’Hebron University Hospital, Barcelona, Spain; Faculty of Medicine, Saarland University Hospital and Saarland University, Homburg, Saarland, Germany; Neurology Department, Hospital Universitari de Girona Doctor Josep Trueta, Girona, Spain; Cardiology Department, Hospital Universitari de Girona DC Josep Trueta, Girona, Spain; Neurology Department, Hospital de Navarra, Pamplona, Navarra, Spain; Cardiology Department, Hospital de Navarra, Pamplona, Navarra, Spain; Neurology Department, Puerta de Hierro Majadahonda University Hospital, Majadahonda, Madrid, Spain; Cardiology Department, Puerta de Hierro University Hospital of Majadahonda, Majadahonda, Madrid, Spain; Department of Neurology and Stroke Center, Hospital La Paz Institute for Health Research-IdiPAZ, Madrid, Spain; Department of Cardiology, Hospital La Paz Institute for Health Research-IdiPAX, Madrid, Spain; Neurology Department, Hospital Universitario de Basurto-OSI Bilbao, Bilbao, País Vasco, Spain; Cardiology Department, Hospital Universitario de Basurto-OSI Bilbao, Bilbao, País Vasco, Spain; Neurology Department, Complejo Hospitalario Torrecardenas Servicio de Neurologia, Hermandad donantes de sangre S/N Almeria, Spain; Cardiology Department, Complejo Hospitalario Torrecardenas Servicio de Cardiologia, Hermandad donantes de sangre S/N Almeria, Spain; Neurology Department, Hospital Universitario Lucus Augusti, Lugo, Galicia, Spain; Cardiology Department, Hospital University Lucus Augusti, Lugo, Galicia, Spain; Neurology Department, Hospital General Universitario de Albacete, Albacete, Castilla–La Mancha, Spain; Cardiology Department, Hospital General Universitario de Albacete, Albacete, Castilla–La Mancha, Spain; Neurology Department, Donostia University Hospital, Donostia, Gipuzkoa, Spain; Cardiology Department, Donostia University Hospital, Donostia, Gipuzkoa, Spain; North West Anglia NHS Foundation Trust, Neurology and Cardiology Department, Edith Cavell Campus, Peterborough, Cambridgeshire, UK; Cardiology Department, University Hospital Centre Zagreb, Zagreb, Croazia

**Keywords:** Echocardiography, stroke, FoCUS, sources of stroke, akinesia, left ventricle dysfunction

## Abstract

**Introduction:**

Focused cardiac ultrasound (FoCUS) has a high diagnostic yield and a rapid theoretical learning curve. FoCUS can be applied in stroke assessments performed by stroke neurologists when a cardioembolic stroke is suspected.

**Patients and methods:**

An international multicenter, prospective validation study was conducted to assess neurologists’ ability to perform FoCUS. The FoCUS examination was defined as a simplified 2D transthoracic echocardiography. Neurologists and cardiologists performed the FoCUS independently and blinded. A twenty-question test evaluated neurologists’ ability to recognize sources of cardioembolic stroke from recorded FoCUS studies.

**Results:**

A total of 432 paired studies involving 216 patients were conducted across 11 centers. No significant differences were found between neurologists and cardiologists in detecting: Left Ventricle (LV) dysfunction (7.4% vs 7.9%, *p* = 0.834), LV dilation (2.8% vs 2.3%, *p* = 0.766), VC collapsibility (7.2% vs 9.1%, *p* = 0.501), Right Ventricle dysfunction (0.9% vs 0.9%, *p* = 0.999), and pericardial effusion (0.5% vs 1.9%, *p* = 0.212). Cohen Kappa showed substantial agreement for LV dysfunction (0.640), moderate for LV dilation (0.589), and fair for VC collapsibility (0.226). Neurologists demonstrated 93.82% sensitivity and 92.92% specificity for detecting embolic sources. Success rate for LV akinesia was 88% (16/18), LV dysfunction 83% (15/18), complex aortic plaque 88% (16/18), and mitral stenosis 55% (10/18).

**Discussion and conclusion:**

Properly trained neurologists can reliably perform FoCUS, particularly for assessing LV function and dilation, with better results in patients with favorable echocardiographic windows. While VC assessment requires further training, neurologists demonstrated high accuracy in identifying cardioembolic sources (over 90% of cases correctly identified). This study supports implementing standardized FoCUS training for neurologists through collaboration with cardiology specialists to enhance stroke diagnostics and management.

## Graphical Abstract

**Graphical abstract:**
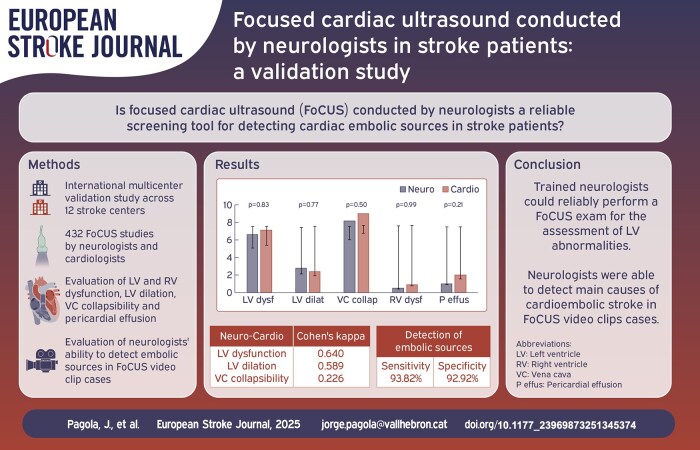

## Introduction

Echocardiography is highly recommended in stroke clinical guidelines because up to 30% of ischemic strokes can have an embolic origin.^1^ Excluding atrial fibrillation, the main sources of emboli are left ventricle (LV) wall motion abnormalities including aneurysms; patent foramen ovale (PFO) in young patients; and severe aortic atheroma plaque.^2–4^ The diagnosis of the cause of the stroke should be made as soon as possible since the risk of recurrence is higher during the first week after the stroke or TIA.^5^ This is particularly important in detecting intraventricular thrombi or in aortic plaques with mobile thrombi due to the higher risk of embolization.^6,7^

There are several diagnostic techniques capable of detecting a cardioembolic source of a stroke. However, these methods are sometimes semi-invasive (transesophageal echocardiography) or have an associated exposure to ionizing radiation (cardiac CT), or a limited availability (cardiac MRI).^8,9^ Therefore, patients benefit from a preliminary test as a screening tool such as transthoracic echocardiography examination (TTE).

Focused cardiac ultrasound (FoCUS) refers to the use of TTE performed by the physician in charge of the patient, with the aim of answering specific clinical questions that seek to provide a diagnostic orientation regarding relevant health problems. This type of study has a high diagnostic yield and a rapid theoretical learning curve.^10^ It is integrated into the clinical decision-making and orientation tree in the diagnosis and treatment of patients because it is indicated in specific clinical situations, mainly defined by specific clinical scenario.^11^ The technical requirements in FoCUS are simplified as just two-dimensional (2D) image and color mode are needed. It should not replace a comprehensive TTE study when abnormal findings suggest relevant heart disease.

Ultrasound techniques are commonly used by stroke neurologists, as they frequently conduct carotid and/or transcranial duplex examinations to identify extra/intracranial stenosis. FoCUS can be applied in stroke assessments as a streamlined TTE performed by stroke neurologists when a cardioembolic stroke is suspected, serving as a screening tool for more advanced diagnostic evaluations such as TEE and cardiac CT. Previous studies have demonstrated that a trained neurologist can accurately perform a TTE study, comparable to a cardiologist’s evaluation.^12^ A similar study showed high sensitivity and specificity in detecting the main causes of cardioembolic stroke, reducing the average patient stay, and implying cost savings for public health.^13^

This study aimed to validate neurologists’ ability to perform FOCUS examination and to detect sources of stroke embolism.

## Patient and methods

This was an international multicenter, prospective, validation study involving consecutive enrollment of acute stroke patients admitted to the stroke Units of collaborating centers.

### Neurologist performance in FoCUS

A concordance validation study was conducted to assess neurologists’ ability to perform FoCUS, the first objective of the study. Neurologists and cardiologists independently performed the FoCUS protocol, unaware of each other’s results, within a 3-day period. Each participating center selected cardiologists based on their expertise in clinical echocardiography and involvement in stroke patient care. The cardiologist’s FoCUS served as the gold standard. The thoracic window quality for FoCUS assessment was noted.

#### FoCUS protocol

The FoCUS examination was defined as a simplified 2D TTE examination with color Doppler code assessment. Left and right ventricle systolic function and size were estimated by eyeballing. Therefore, measurements of chamber dimensions and systolic function by ejection fraction formula (Simpson) were not permitted. Neither pulsed wave analysis nor continuous Doppler were allowed.

To perform the FoCUS study the patients were invited to lie in both supine and lateral decubitus positions. The suprasternal view; parasternal long and short axis view; apical four, five and three chambers view; and subcostal long and short axis view were explored, following international recommendations.^10^ Color coded was employed to detect mitral or aortic valvulopathies. The type and the model of each echo machine was not restricted but pocket size echo was not allowed.

To record the answers for the FoCUS examination, we employed a modified report previously used by non-cardiologists to evaluate bedridden patients with neurological diseases.^14^ The report contained five yes/no questions to assess left ventricular function, size, the size, collapsibility of the inferior vena cava (VC), right ventricular (RV) function, and presence of pericardial effusion (see Appendix 1). The five-question FoCUS protocol was designed to balance comprehensiveness with feasibility for non-cardiologists. LV dysfunction and dilation were included as they represent common findings in cardioembolic stroke. VC collapsibility and RV dysfunction provide information about volume status and right heart pressures, which can impact management decisions. Pericardial effusion, while not directly related to cardioembolic stroke, is an important finding that may require urgent management and impact antithrombotic therapy decisions. LV akinesia and wall motion abnormalities were evaluated as part of the LV dysfunction assessment in our protocol.

#### Training program in FoCUS

A training program for neurologists (FoCUS training) was carried out from 2021 to 2023 with the completion of more than a 100 FoCUS studies in 12 hospitals in Spain, the United Kingdom and Germany. The training in FoCUS in Spain followed a published protocol developed after the 2018 agreement between the Spanish Society of Neurology and the Spanish Society of Cardiology. This protocol requires at least 100 Focus examinations of supervised real-world training with patients in a variety of clinical situations. The same protocol has been implemented in the participating centers in the United Kingdom and Germany.^15^ In detail, the neurologist performed up to 50 studies under the supervision of the certified cardiac imaging cardiologists and 50 more studies under the supervision of coordinators and previously trained neurologists (JP, RM, and AR) totaling 100 supervised FoCUS examinations before independent practice.

Training with the cardiologists focused on capturing and interpreting echocardiography planes, while sessions with the neurologist aimed at identifying embolic sources in echocardiographic images. Neurologists learned to estimate left ventricular (LV) systolic function by eye balling observation, estimate left ventricular size, detect pericardial effusion, and VC collapsibility. During training, all cases of ventricular LV akinesia, LV dysfunction, mitral stenosis, or detected aortic plaque were documented.

#### Patients and centers

All patients underwent standard diagnostic work-up including neuroimaging (CT/MRI) to confirm cerebral infarction, CT-angio or Doppler to assess vessel stenosis, and EKG/cardiac monitoring to detect atrial fibrillation or flutter.

The study included patients over 18 years of age with acute ischemic stroke or TIA who were admitted to the 12 stroke units managed by the trained neurologists. Patients with atherothrombotic strokes were excluded, as well as patients with previously known causes of stroke, not requiring echocardiography for work-up (e.g. carotid dissection, atrial fibrillation), poor short-term prognosis, and those who declined participation.

### Neurologist ability to detect cardioembolic sources of stroke by FoCUS

The second objective of the study was to evaluate the ability of a trained neurologist to recognize sources of cardioembolic stroke by reviewing recorded FoCUS studies.

A 20-question test with anonymized images was given. The echocardiographic video clip included suprasternal, parasternal, apical, and subcostal views. The neurologist was asked to determine if a stroke source was present in the clip (yes/no question) and specify the type of source observed in each clip (one correct answer among four possible options). There were two cases each of LV apical akinesia, LV dysfunction, mitral stenosis, and aortic plaque.

### Data protection and ethical

All participants provided informed consent, compliant with Law 3/2018 on Personal Data Protection and Regulation (EU) 2016/679. The study followed the Declaration of Helsinki (2008), good clinical practice standards, and relevant legal regulations. No data except for echocardiographic images was allowed to be collected for the purpose of the study.

### Statistical analysis

For the concordance validation study the descriptive analysis of the questionnaire results following the S-FOCUS protocol assessed the completion rate. A concordance study using Cohen’s Kappa index (*K* = Po − Pe/1 − Pe) quantified the agreement level between two evaluators for each protocol item with its respective 95% confidence interval (CI). Given the low interrater agreement in ultrasound examination a kappa concordance index threshold of over 0.6 was considered acceptable.^16^ The sample size estimation was derived from analogous studies that validated echocardiographic measurements by recruiting a minimum of 78 patients. Accounting for up to 20% of inconclusive cases due to suboptimal thoracic windows, a minimum sample size of 94 patients per group (*n* = 186 studies) was recommended to achieve a statistical power of 80% with a significance level of 5%. McNemar Test assessed the impact of discordant pairs due to the disproportionate representation of the cases. STARD recommendations were followed to show agreement in FoCUS between neurology and cardiology.^17^

Neurologists’ accuracy in diagnosing sources of cardioembolic stroke by evaluating clip video cases was evaluated for the second objective. A true positive was defined as correctly identifying pathological cases, while a false negative indicated an incorrect identification. Similarly, a true negative referred to correctly identifying normal cases, and a false positive indicated an incorrect identification. Sensitivity and specificity were then calculated based on these definitions with its respective 95% confidence interval (CI).

The analyses were conducted using Stata 15.1, SPSS 21, R 3.6.1, or the most recent versions available at the time of analysis.

## Results

### Neurologist performance in FoCUS

A total of 432 paired studies involving 216 patients examined by neurologists and cardiologists were conducted across 11 centers from July 2024 to December 2024. One center did not include any patients due to the lack of a participating cardiologist to validate the Focus results. A suboptimal thoracic window was observed in 14.8% (32/216) of neurology cases and 17.6% (38/216) of cardiology cases (*p* = 0.358).

**Figure 1.**
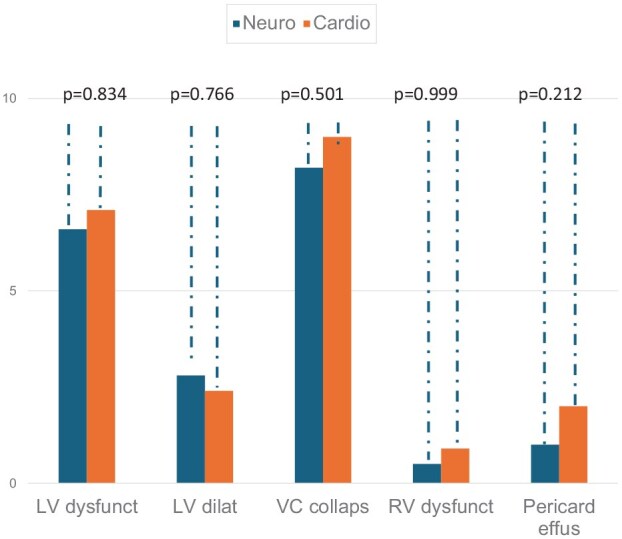
Percentage of patients according to each question on the FoCUS validation questionnaire. LV dysfunct: left ventricle dysfunction; LV dilat: left ventricle dilation; VC collapsa: vena cava collapsibility; RV dysfunct: right ventricle dysfunction; Pericard effus: pericardial effusion.

Figure 1 shows the percentage of patients according to each question on the FoCUS validation questionnaire: LV dysfunction was 7.4% (16/215) for neurologists and 7.9% (17/215) for cardiologists (*p* = 0.834). LV dilation was observed in 2.8% (6/213) of cases for neurologists and 2.3% (5/213) for cardiologists (*p* = 0.766). VC was not collapsible at 7.2% (15/208) and 9.1% (19/208) for cardiologists (*p* = 0.501). RV dysfunction was observed at 0.9% (2/216) for neurologists and 0.9% (2/216) for cardiologists (*p* = 0.999). Pericardial effusion for neurologists 0.5% (1/212) and 1.9% (4/212) for cardiologists (*p* = 0.212). Other relevant findings classified as other findings in the questionnaire are listed in Table 1.

**Table 1.** Percentage of other abnormalities detected by neurologists and cardiologists following FoCUS protocol assessment.

*n* = 213	Neurologist	Cardiologist	*p*-Value
Left atrial enlargement, % (*n*)	24.0% (52)	23.9% (51)	0.851^a^
Mitral stenosis, % (*n*)	3.2% (7)	1.9% (4)	0.530^a^
RV dilatation, % (*n*)	0.5% (1)	1.4% (3)	0.360^b^
Thrombus detection, % (*n*)	0.5% (1)	0.5% (1)	0.999^b^

RV: right ventricle.

^a^ **χ** ^2^ test.

^b^Fisher’s exact test.

#### Agreement between neurologist and cardiologists in FoCUS

**Figure 2.**
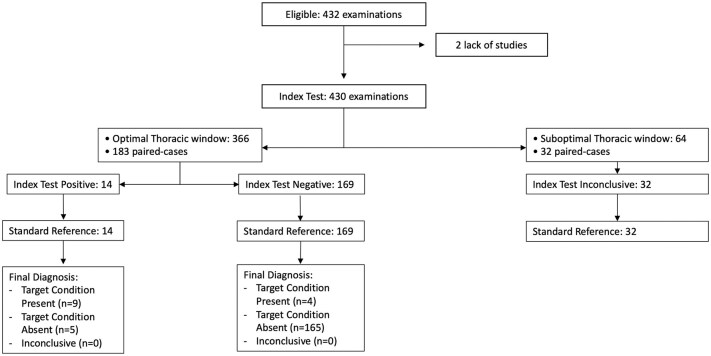
STARD diagram of FoCUS agreement between neurologists and cardiologists for left ventricle (LV) dysfunction evaluation. In patients with optimal thoracic window the agreement was substantial (Cohen Kappa 0.640, 95% CI [0.424–0.855]).

For the FoCUS evaluation of LV dysfunction, 430 out of 432 cases were compared. Two cases were excluded due to lack of cardiological assessment (*n* = 2). Kappa index showed moderate agreement (Cohen Kappa 0.596 [95% CI 0.394–0.786]); In the evaluation of LV dysfunction for neurologists there were 85.5% (183/214) patients with optimal thoracic window and 82.7% (177/214) patients for cardiologists (*p* = 0.428). Considering the patients with optimal thoracic window for neurologists (*n* = 183) the concordance improved significantly (Cohen Kappa 0.640, [95% CI 0.424–0.855]; Figure 2). Inconclusive cases for LV dysfunction evaluation due to suboptimal thoracic window were for neurologists (*n* = 31) and for cardiologists (*n* = 31). No significant differences in overdiagnosis were observed between neurologists and cardiologists, as indicated by a McNemar Test result of 0.999 in patients with an optimal thoracic window.

**Figure 3.**
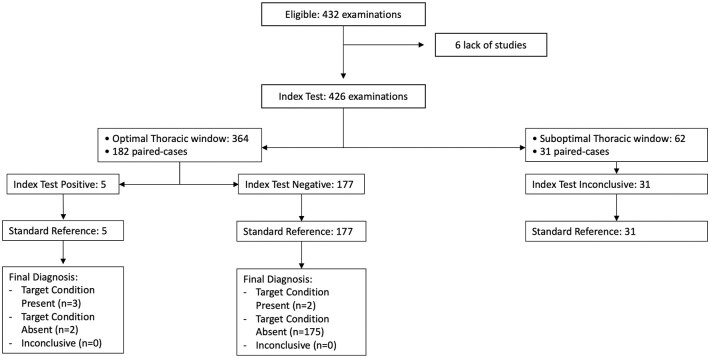
STARD diagram of FoCUS agreement between neurologists and cardiologists to evaluate LV dilation. In patients with optimal thoracic window the agreement was substantial (Cohen Kappa 0.589, 95% CI [0.236–0.941]).

For the evaluation of left ventricular (LV) dilation, a total of 426 out of 432 cases were analyzed. Six cases were excluded due to the absence of either a cardiological assessment (*n* = 3) or a neurological assessment (*n* = 3). The Kappa index indicated moderate agreement, with Cohen’s Kappa at 0.53 and a 95% confidence interval of [0.181–0.886]. Among the patients evaluated for LV dilation, 84.65% (182/213) had an optimal thoracic window as assessed by neurologists, compared to 82.24% (176/213) by cardiologists, with no statistically significant difference (*p* = 0.347). In cases where patients with optimal thoracic windows were evaluated by neurologists (*n* = 182), concordance improved, reflected by a Cohen’s Kappa of 0.589 and a 95% confidence interval of [0.236–0.941] (see Figure 3). Inconclusive cases due to suboptimal thoracic windows were noted for both neurologists (*n* = 32) and cardiologists (*n* = 32). Furthermore, no significant differences in overdiagnosis were found between the two groups (McNemar Test 0.999).

**Figure 4.**
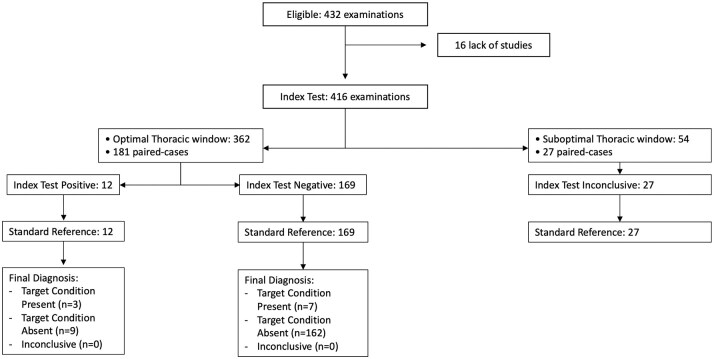
STARD diagram of FoCUS agreement between neurologists and cardiologists for VC collapsibility evaluation. In patients with optimal thoracic window the agreement was slight (Cohen Kappa 0.226, 95% CI [−0.02 to 0.480]).

In the evaluation of VC collapsibility, 416 cases were compared. For the neuro-cardiological agreement (*n* = 208), the Kappa index indicated fair agreement (Cohen’s Kappa 0.296, 95% CI [0.08–0.511]). Considering patients with an optimal thoracic window for neurologists (*n* = 181), the concordance did not improve (Cohen’s Kappa 0.226, 95% CI [−0.02 to 0.480], McNemar Test *p* = 0.804; Figure 4). No overdiagnosis was found between the groups (McNemar Test 0.999).

There were insufficient pathological cases to evaluate the concordance of RV dysfunction and pericardial effusion.

### Neurologist ability to detect cardioembolic sources of stroke by FoCUS

Nine neurologists completed the questionnaire. Out of 20 questions, 180 answers were obtained, of which 99 questions were from recorded studies without embolic source and 81 cases questions with embolic source (see Appendix 2).

For cases with an embolic source, 76 answers were correct and five incorrect. For cases without an embolic source, 92 answers were correct while seven were incorrect. As a result, the neurologist’s performance in detecting embolic sources demonstrated a sensitivity of 93.82% (76/81); 95% CI [85.55–97.71] and a specificity of 92.92% (92/99); 95% CI [85.49–96.87] (Table 2).

**Table 2.** Answers to the question with and without embolic source. FoCUS recorded studies to detect stroke cardioembolic sources. Twenty questions to nine neurologists with nine pathological cases and 11 normal cases.

Questions test	Questions with embolic source	Questions without embolic source	Total
Answered as embolic source	76	7	83
Answered as non-embolic source	5	92	97
Total	81	99	180

Regarding the identification of the type of embolic source two cases for each embolic source were shown. The nine neurologists provided a total of 18 responses per case. The mean success rate for each embolic source was as follows: LV akinesia 88% (16/18), LV dysfunction 83% (15/18), complex aortic plaque 88% (16/18), and mitral stenosis 55% (10/18).

## Discussion

This multicenter international study represents the largest validation to date of neurologist-performed FoCUS in stroke patients. While previous single-center pilot studies have explored this concept, our study is distinctive in its comprehensive methodology, including power calculation, multicenter design across three countries, and dual validation approach examining both technical performance and diagnostic accuracy.

The first objective of the study was to demonstrate the neurologist’s ability to perform and interpret images of FoCUS. Mastering the conduct and interpretation of the technique is the initial step to perform an accurate assessment with ultrasound-based techniques.^18^ Our results demonstrated no significant difference between neurologists and cardiologists in detecting pathological cases of left or right ventricular dysfunction, pericardial effusion, and vena cava collapsibility. However, it should be noted that these events were infrequently observed. The concordance validation study showed that properly trained neurologists could reliably perform a FoCUS exam, particularly for the assessment of LV function and LV dilation. This reliability increases when the patient has a favorable echocardiographic window. Therefore, for neurologists’ evaluations using FoCUS caution should be exercised in cases with suboptimal windows. However, even in cases with optimal window LV dysfunction and LV dilation were not detected in 2.73% (5/183) and in 1.12% (3/182) respectively. Thus, it is advisable to review the patient’s full medical history, including prior conditions, diagnostic tests, and clinical examinations, to assess findings more accurately.^19^

Conversely, evaluating VC collapsibility necessitates further training for neurologists, as the concordance was low and did not improve even after excluding poor thoracic windows. Evaluating VC is more accurate with VC diameter measurements, which were not included in our protocol.^20^

While valuable data on the LV and VC were obtained, there was insufficient information on pericardial effusion and the RV dysfunction. This was due to the consecutive enrollment of the stroke cases, who are less likely to have these conditions, making them more relevant to heart disease assessments rather than those for stroke patients.^21^

Other abnormalities were identified by FoCUS, with no differences observed by neurologists and cardiologists in the detection rates of left atrial enlargement, mitral stenosis, right ventricle dysfunction, and intracardiac thrombus. Since it was recorded as other findings, it cannot be assured that it was evaluated equally in all patients. Therefore, a concordance test was not considered for these alterations, although the detection rate was recorded for each group. Except for left atrial enlargement the occurrence rate was so low that a different study design would be required to evaluate them accurately.

The second objective was to evaluate trained neurologists’ ability to detect cardioembolic sources reviewing images of FoCUS studies. Due to the low prevalence of embolic stroke sources, a questionnaire-based study reviewing video clip of FoCUS studies was performed. Tests were conducted including cases with and without embolic sources. The data showed that neurologists identified a high percentage of cases without embolic sources and those with the primary embolic sources. This second objective also examined success rates for specific embolic sources. Neurologists detected LV dysfunction and ventricular akinesia in over 80% of cases. Although identifying segmental contractility disorders typically requires expert echocardiography, neurologists demonstrated high proficiency in recognizing LV akinesia (88% success rate). This finding is clinically significant as it often triggers comprehensive cardiological evaluation due to its association with thrombus formation, though just one cardiac thrombi was detected in our study population. However, the goal of the FoCUS assessment is to address a clinical question related to the absence of movement in the LV, which may be easier for a neurologist to identify. When akinesia is identified using FoCUS, it is advisable to conduct a comprehensive transthoracic echocardiography to evaluate ischemic heart disease.^22^

Aortic plaques were also detected in the aortic arch through FoCUS assessment. Although FoCUS is not the most reliable method for detecting aortic plaques due to its limited sensitivity and specificity, it provides an accessible evaluation tool for the aortic arch.^23^ Moreover, FoCUS can assist in selecting cases for further assessment with transesophageal echocardiogram or aortic CT to better evaluate aortic plaques.

The detection rate of mitral stenosis was lower compared to other conditions. The findings indicate that advanced study using Doppler velocimetry is required to confirm the presence of stenosis in cases of mitral pathology. Although mitral stenosis may be identified using the Focus protocol, it is not considered suitable for assessing severity.^24^ Therefore, comprehensive echocardiographic evaluation is mandatory.

Our results could lead to recommendations for other societies to implement similar diagnostic protocols in their centers. The integration of FoCUS into neurological practice is an evolving field aimed at enhancing diagnostic and management strategies for patients with neurological disorders. This approach is especially pertinent in stroke care, where identifying cardiac sources of embolism is critical. FoCUS practice aligns with the broader application of ultrasonography in neurology, which provides real-time, cost-effective, and radiation-free diagnostic advantages.

While a comprehensive initial echocardiographic examination might seem ideal, the reality in many healthcare systems is limited timely access to cardiologist-performed studies. The FoCUS approach enables rapid bedside screening by the neurologist, allowing for immediate triage decisions. This two-step approach may optimize resource utilization by reserving comprehensive studies for patients with abnormal FoCUS findings while maintaining diagnostic accuracy. Future research should include cost-effectiveness analyses comparing these approaches across different healthcare settings.

Neurologists performing FoCUS should undergo accredited training to ensure proficiency in the technique. While the use of FoCUS by neurologists is promising, it is essential to validate a protocol for comprehensive training and collaboration with cardiology specialists to ensure accurate diagnosis and effective management. The European Association of Cardiovascular imaging designed the curriculum and syllabus to be trained in FoCUS.^25^ The integration of FoCUS into neurology requires collaboration and supervision from cardiac imaging units to optimize patient outcomes. In 2018, an agreement between the Spanish Society of Neurology and the Spanish Society of Cardiology facilitated the training and certification of neurologists in FoCUS. In Spain, neurologists seeking FoCUS certification must complete an accredited training program and pass a certification exam through their professional society, which involves a modest financial investment. The 100 training FoCUS studies are typically expected to be collected over a period of 3 months, and while they presents a logistical challenge, it’s important to note that these training cases have broader inclusion criteria than our validation study. In 2019, the European Society of Neurosonology established an expert ultrasonography group to advance the FoCUS concept in stroke diagnosis. The Focused Echocardiography Study Group initiated efforts to disseminate and implement FoCUS practices across various regions. The intention of the working group is to enable interested neurologists and non-cardiologist physicians involved in the care of stroke patients to learn FoCUS skills and is committed to develop a training program based on these findings.

The aim of FoCUS protocol is to answer if there are sources of cardioembolic stroke. Focus should be ordered just for cardiac work up in stroke, if patients have other symptoms, such as dyspnea or angina, they should not undergo FoCUS but standard echocardiography in expert hands. Currently in Spain, more than 25 neurologists across 15 stroke units have been trained and certified in the FoCUS protocol to date, and regularly employ FoCUS as part of their stroke assessment protocol. Implementation varies by region and institution, with ongoing efforts to expand training opportunities through national societies. The successful implementation in these centers demonstrates the feasibility of this approach in real-world clinical settings. To ensure the protocol is effectively implemented in all Centers, it is recommended that at least two stroke neurologists from each Institution receive training to provide backup support.

There is a potential risk that certain cardiac pathologies might be overlooked when the neurologist does not find abnormalities on FoCUS examination. However, this risk is mitigated by the high sensitivity (93.82%) demonstrated by neurologists in identifying embolic sources. Additionally, the clinical context and other diagnostic findings guide the decision for further cardiological evaluation. Future studies should evaluate the long-term outcomes of patients with negative FoCUS findings by neurologists to determine if clinically significant pathologies are being missed.

This study has certain limitations. A variety of ultrasound devices were used across the participating centers. This heterogeneity was not included in the analysis due to the significant variability across different centers. In most centers, cardiologists had access to higher-specification devices compared to neurologists, which may have created a bias against neurologist performance. However, this variability reflects real-world clinical practice and enhances the generalizability of our findings. Additionally, some questions in the FoCUS report were challenging to answer without specific measurements, potentially decreasing the concordance between evaluators. Since only echo images were collected for the purpose of this study, further analysis concerning patient age, comorbidities or stroke characteristics was not conducted. Furthermore, the questionnaire-based assessment of embolic source detection is limited by the absence of a control group of untrained neurologists or external cardiologists. Future studies should include larger numbers of evaluators with varying levels of training to better define the learning curve and optimal training requirements. Analysis of the correlation between FoCUS findings and comprehensive echocardiography was beyond the scope of this validation study but represents an important area for future research, as a comprehensive TTE includes assessment of multiple cardiac parameters that fall outside the scope of practice for stroke neurologists.

## Supplementary Material

sj-docx-1-eso_23969873251345374

## Data Availability

The datasets generated and analyzed during the current study are not publicly available due to privacy regulations and restrictions related to patient confidentiality but are available from the corresponding author upon reasonable request.

## ORCID iDs

Giulio Maria Fiore   https://orcid.org/0009-0005-4055-984X

Felipe A Montellano   https://orcid.org/0000-0002-9438-0854

Victor Gonzalez   https://orcid.org/0000-0001-7243-0876

Juan Alvarez-Cienfuegos   https://orcid.org/0000-0003-1604-2179

Alberto Vera   https://orcid.org/0000-0003-2181-0961

Angel Ruiz   https://orcid.org/0009-0002-2924-2057

Jesús González Mirelis   https://orcid.org/0000-0002-3083-8627

Juan Manuel García-Sánchez   https://orcid.org/0009-0007-0237-8313

Laura Amaya Pascasio   https://orcid.org/0000-0003-1162-4209

Vlatka Reskovic   https://orcid.org/0000-0002-4721-3236
