# Supplementary material for: Focused cardiac ultrasound conducted by neurologists in patients with stroke: A validation study
Source: Eur Stroke J. 2026 Jan 1;11(1):23969873251345374. doi: 10.1093/esj/23969873251345374 (PMC12866232; doi:10.1093/esj/23969873251345374)
Supplement: sj-docx-1-eso_23969873251345374 [file sj-docx-1-eso_23969873251345374.docx]

### Supplemental Material

#### Appendix 1

The S-FOCUS protocol involves a focused echocardiography study to answer the following questionnaire:

1. Is the left ventricular function impaired (EF <50% by visual estimation)? (yes/no)
2. Is the left ventricle dilated? (yes/no).
3. Is the vena cava collapsable? (yes/no)
4. Is the right ventricular function impaired? (yes/no)
5. Is there a pericardial effusion? (yes/no)
6. Other abnormalities? (open question)

#### Appendix 2

FOCUS video clip case 1: Complex Aortic Arch Plaque

FOCUS video clip case 2: Apical Akinesia

FOCUS video clip case 3: Left Ventricular Dysfunction

FOCUS video clip case 4: Mitral Stenosis
